# The Stability of a Nanoparticle Diamond Lattice Linked by DNA

**DOI:** 10.3390/nano9050661

**Published:** 2019-04-26

**Authors:** Hamed Emamy, Oleg Gang, Francis W. Starr

**Affiliations:** 1 Department of Physics, Wesleyan University, Middletown, CT 06459, USA; hemamy@wesleyan.edu; 2 Department of Chemical Engineering, and Department of Applied Physics and Applied Mathematics, Columbia University, New York, NY 10027, USA; ogang@bnl.gov; 3 Center for Functional Nanomaterials, Brookhaven National Laboratory, Upton, NY 11973, USA; 4 Department of Molecular Biology & Biochemistry, Wesleyan University, Middletown, CT 06459, USA

**Keywords:** DNA nanotechnology, self-assembly, nanoparticle superlattice

## Abstract

The functionalization of nanoparticles (NPs) with DNA has proven to be an effective strategy for self-assembly of NPs into superlattices with a broad range of lattice symmetries. By combining this strategy with the DNA origami approach, the possible lattice structures have been expanded to include the cubic diamond lattice. This symmetry is of particular interest, both due to the inherent synthesis challenges, as well as the potential valuable optical properties, including a complete band-gap. Using these lattices in functional devices requires a robust and stable lattice. Here, we use molecular simulations to investigate how NP size and DNA stiffness affect the structure, stability, and crystallite shape of NP superlattices with diamond symmetry. We use the Wulff construction method to predict the equilibrium crystallite shape of the cubic diamond lattice. We find that, due to reorientation of surface particles, it is possible to create bonds at the surface with dangling DNA links on the interior, thereby reducing surface energy. Consequently, the crystallite shape depends on the degree to which such surface reorientation is possible, which is sensitive to DNA stiffness. Further, we determine dependence of the lattice stability on NP size and DNA stiffness by evaluating relative Gibbs free energy. We find that the free energy is dominated by the entropic component. Increasing NP size or DNA stiffness increases free energy, and thus decreases the relative stability of lattices. On the other hand, increasing DNA stiffness results in a more precisely defined lattice structure. Thus, there is a trade off between structure and stability of the lattice. Our findings should assist experimental design for controlling lattice stability and crystallite shape.

## 1. Introduction

Nanoparticle (NP) assemblies have numerous demonstrated and predicted applications, such as optical or electronic materials, biosensing, drug delivery and others [1,2,3,4]. For many of these applications, an ordered structure of NPs, rather than an amorphous structure, is preferred or even necessary [5,6,7,8]. The order of NPs can be controlled by functionalizing NPs with polymers or DNA. The specific recognition and sequence control offered by DNA has made it a particularly useful choice. In this approach, NPs are functionalized with DNA that have “sticky ends” where single-stranded (ss) DNA can hybridize to form a bond that directly links NPs [9,10,11,12,13]. The crystallization of particles mediated by DNA interactions was first realized in micron-scaled colloidal particles [14], and subsequently for NPs [15,16]. Alternatively, DNA origami methods have also been used to create 2d or 3d structures [3,17,18] that serve as building block for the lattices [19,20,21,22,23].

There have been significant advances in creating NP lattices with a broad range of lattice symmetries and lattice spacings [12,13,15,16,24]. Because the NPs are usually functionalized uniformly, their interactions are correspondingly isotropic. As a result, the lattices realized correspond to close-packed structures where NP have eight or more nearest neighbors. Creating ordered structures for open lattices (coordination number less than 6) has proved more challenging. In particular, one important symmetry that proved elusive for NP assembly strategies is the diamond lattice. Moreover, cubic diamond (CD) lattices with nm scale spacing are predicted to have interesting optical properties [4,25], such as a complete optical band gap in 3D [5]. In traditional atomic and molecular systems, such low coordination lattices require directional interactions. Although theoretical and computational studies have shown it is possible to create a cubic diamond lattice by an isotropic potential [26,27], creating such a potential using uniformly functionalized NPs can be challenging. To overcome the challenge of assembling low-coordination lattices, we combined the DNA origami method with NPs to create DNA-functionalized NPs with directional interactions that are able to self-assemble into CD symmetry [28], opening the possibility to realize these novel materials. The methods to create NPs with customizable directional interactions is an area of ongoing research [29,30]. Other experimental methods of synthesizing CD lattices have been proposed [31,32]. While in this work we use direct DNA hybridization between origami and DNA-functionalized NP to create a 3D ordered structure, DNA origami can be used in different ways to create ordered structure of NPs (such as using electrostatic interactions between NPs and DNA origami rather than DNA hybridization [33], or creating an ordered 3D framework from DNA origami to host NPs [34]).

In order to better control the properties of diamond structured NP lattices, we need an improved understanding of the factors that control lattice stability and crystallite shape. NP size can potentially alter both the lattice stability as well as the optical properties of lattices, both due to changes in the NP optical properties and changes of the lattice spacing. In this work, we study the relative stability of CD superlattices as a function of NP size and DNA stiffness using molecular dynamics (MD) simulations. We evaluate the relative stability of lattices based on their free energy. We find that entropic effects dominate the changes of free energy. We break the entropic effects into the contributions from the translational and rotational degrees of freedom. We find that the translational entropy stabilizes the lattice as the NP size increases; in contrast, the rotational degrees of freedom have a destabilizing effect as the NP size increases. The contributions to the entropy from the rotational orientation outweigh those of the translational degrees of freedom. We show that we can enhance the lattice stability (lower free energy) by increasing the flexibility of the DNA strands, which in turn, increases the contribution from the rotational entropy. On the other hand, increasing the flexibility results in a less well-defined structure. Thus, from a design perspective, there is a balance between enhancing stability and optimizing the desired order. The important role played by entropy in diamond packings has been recognized previously [35,36]. For example, Ref. [35] showed that the degree of truncation of tetrahedra, similar to the functionalized NP we study, is central to the packing (entropic effects) that determine the stability of the CD lattices.

We also evaluate the equilibrium shape of the cubic diamond crystallites using the Wulff construction [37]. The Wulff construction predicts crystallite shape based on the surface free energy. Since the experimental method for making NP lattices is a quasi-static process, the equilibrium shape should correspond to the crystallite shape in experiments [38,39]. In most cases, the simulation results for surface energy can be anticipated from a simple approximation for the number of broken bonds along any crystal face. However, we find that when the DNA are flexible enough to allow reorientation at the surface, alternate bonds form that reduce the surface energy, and the resulting predicted crystallite shape can be different from that expected for atomic systems with diamond structure (e.g., diamond, Si, or SiO${}_{2}$) [40,41,42].

The manuscript is ordered as follows: Section 2 explains the model for DNA, NP and tetrahedral cages and the MD simulation methods. In Section 3.1, we discuss the equilibrium shape of the crystallite, Section 3.2 explains the theory and the results for the relative stability as a function of particle size and DNA stiffness. We briefly conclude in Section 4.

## 2. Materials and Methods

A four-coordinated CD lattice typically requires highly directional interactions. Experimentally, directional NP interactions were realized by placing a spherical NP in the middle of a tetrahedral cage made by DNA origami methods [28]. These directional NPs are combined with isotropically DNA-functionalized NPs to create CD lattices. To determine the relative stability of CD lattices created using this approach, we use a simple coarse-grained model to mimic the experimental system studied in ref. [28]. The details of this model are described in the following.

### 2.1. Tetrahedral Cage Model

**Figure 1:**
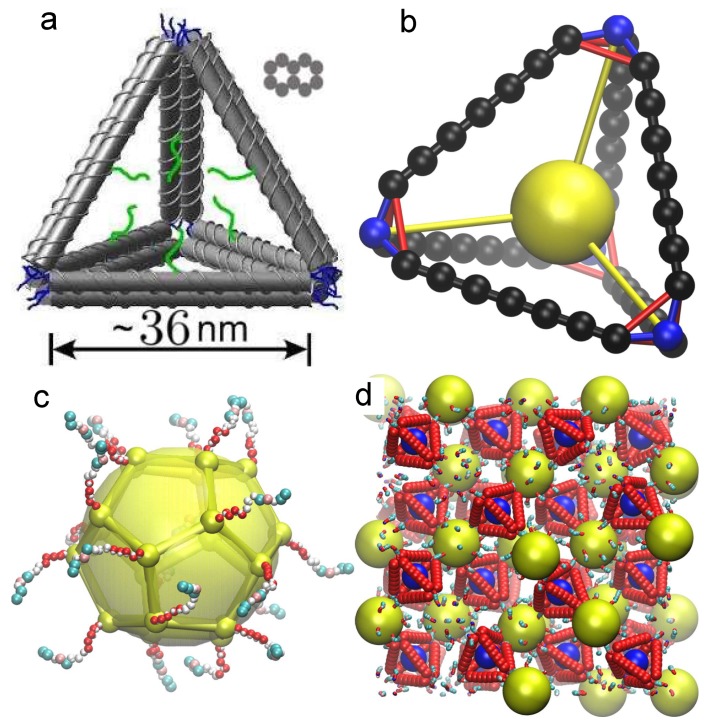
(**a**) Schematic of the tetrahedral cage used in previous experiments [28]. Each dsDNA in the tetrahedral edge is represented by gray cylinder. The figure also shows the cross-section of an edge to indicate relative positions of ten dsDNA bundles. The blue lines indicate ssDNA linkers at the vertices, and the green lines are the ssDNA linkers used to attach a NP at the cage core. (**b**) Schematic of tetrahedral cage bond structure used in the MD simulation. The spheres show the particle positions and the lines show the bonds between particles. Different colors shows different bonds explained in the main text. The sphere sizes are not representative of the actual size of particles. (**c**) The schematic of NP used in the MD simulation. Small spheres show the position of the vertices, and the large sphere shows the central particles and the lines show the bonds between vertices. (**d**) A simulation snapshot of the full lattice structure; the edges appear “ragged” owing to the representation of periodic boundary conditions.

We first describe how we mimic the tetrahedral DNA origami “cages” that were experimentally used to create tetra-functional NP; see Figure 1a for a schematic of the experimental system. Experimentally, the edges of the tetrahedral cage are constructed from “bundles” of 10 double-stranded (ds) DNA that are linked together to form relatively rigid “beams” [28]; the cross-section of these bundled beams is roughly rectangular. Since the individual DNA strands play no functional role in the edges other than structural integrity, in our coarse-grained model we simplify by representing each edge of the tetrahedral cage by seven beads with a diameter comparable to that of the dsDNA bundles (Figure 1b); specifically, the diameter of the particles corresponds to average of the side of a bundle cross-section. Each tetrahedral site on the edge of the tetrahedral (black beads in Figure 1b) is described by a Weeks–Chandler–Andersen (WCA) potential with particle diameter 9.9σ. Following [43], the diameter can be approximately mapped to real units with σ≈0.65 nm. Particles along the edge are linked by a finitely extensible nonlinear elastic (FENE) potential

(1) $$V_{\mathrm{FENE}}=-\frac{k_{b}R_{0}^{2}}{2}\ln\left[1-{\left(\frac{r-\Delta }{R_{0}}\right)}^{2}\right]+4\epsilon \left[{\left(\frac{\sigma }{r-\Delta }\right)}^{12}-{\left(\frac{\sigma }{r-\Delta }\right)}^{6}\right]+\epsilon .$$

For the standard FENE potential, Δ=0, σ=1, and $R_{0}$ is the maximum bond length. Here, Δ is a shift in the equilibrium bond length and $R_{0}+\Delta$ is the maximum bond length. See Table 1 for parameters.

To make the edges more rigid, next-nearest neighbor edge particles are also bonded via a FENE potential with a bond length chosen to prefer linearity among triplets. As Figure 1b shows, the vertices of the tetrahedral cage are truncated, and consist of three particles, tethered to a fourth bead (blue bonds in Figure 1b) that represents the first nucleotide of the DNA linker connected to the tetrahedron. The three vertex beads are linked both to each other (red bonds in Figure 1b), and to edge beads of the cage. Finally, there is a particle at the center of the tetrahedral cage that is described by a WCA potential with particle diameter of 40σ. To improve the rigidity of the cage structure, we include a bond between the first bead of the DNA at the vertex and the particle at the center of tetrahedron (yellow bonds in Figure 1b). The details of these interactions and their coefficients are provided in Table 1.

In order to have a tetrahedral edge that mimics the rod-like nature of the tetrahedral edges in the experiment, we choose the bond length (7.32σ) between edge monomers somewhat smaller than the size of the excluded volume interactions (9.9σ) of these monomers, so that their exclusion zones overlap. For nearest neighbor edge monomers, there are no non-bonded interactions to avoid large values of the pair interactions due to this overlap. At each vertex, there is a ssDNA attached to the tetrahedron. The DNA model is described in a subsequent section. This DNA has two distinct regions: a spacer region of seven nucleotides that has no complement, and a “sticky” region of eight nucleotides that are complementary to the ssDNA attached to NP. The DNA sequence is chosen in such a way that it can only connect with the DNA of NPs, and cannot bond to the DNA of the other tetrahedra. To ensure that DNA strands are roughly normal to the vertex of the tetrahedron, we use an angle bond potential $V_{\mathrm{angle}}=k_{a}\left(1+\cos(\theta )\right)$ where θ is the angle made between the DNA site at the vertex of the tetrahedron with the center of tetrahedron and the next DNA site. The strength of the stiffness, $k_{a}$, varies between 0.5 and 2.0 for different systems similar to the DNA stiffness. Finally, there is a force site at the core of the tetrahedron to ensure excluded volume with the strands, which we describe in the section on pair interactions of the DNA.

### 2.2. Nanoparticle Model

To mimic the polyhedral shape of a typical nanoparticle, we model a NP by a collection of 20 force sites located at the vertices of a dodecahedron. Each dodecahedral site is also described by the WCA potential used for tetrahedral sites, but with diamater σ=1. In addition, there is a force site at the center of the dodecahedron, for a total of 21 force sites for each NP. Each vertex site is bonded via the FENE potential to the nearest neighbor vertices and to the center of the dodecahedron. The bonds are stiff so that the NP is rigid and maintains its shape during the simulation. Since the sites on the vertices are small relative to the dodecahedron, we include an excluded volume interaction with the NP center with a range corresponding to the inscribed sphere of the dodecahedron (see Table 2). There are ssDNA attached to each vertex, for a total of 20 ssDNA attached to each NP. Each DNA strand consists of 15 nucleotides: seven spacers and eight linkers. The sequence for the sticky ends of DNA is chosen so that NP can only bond with a tetrahedral cage and cannot bond to other NPs. The values for FENE bonds are given in Table 1. To ensure that DNA strands are roughly normal to the vertex of the dodecahedron, we use an angle bond potential $V_{\mathrm{angle}}=k_{a}\left(1+\cos(\theta )\right)$ where θ is the angle made between the DNA site at the vertex of the NP with the center of NP and the next DNA site; these angle interactions are identical to those used for the DNA attached to the tetrahedral cages.

### 2.3. DNA Model

For DNA, we use a simple two-site coarse-grained model that was previously developed to describe DNA-functionalized NP assemblies [28,43,44]. In this model, each DNA nucleotide consists of two force sites: (i) the sugar and phosphate back-bone and (ii) the nucleobase site. The backbone sites are bonded to each other, as well as to a nucleobase with a FENE bond to create ssDNA. The only attractive interactions are between nucleobases (A-T or C-G) of distinct ssDNA strands, which leads to the formation of dsDNA. For details of this model, please refer to refs. [43,44,45]. We present most results in Lennard–Jones (LJ) reduced units, where the unit of length is given in terms of σ (diameter of the DNA backbone beads), temperature is in units of $\epsilon /k_{B}$, and time is in the units of $\sigma \sqrt{m/\epsilon }$.

The pair interactions between different units—sites of the tetrahedral cages, NPs, or DNA—are given by an expanded WCA potential

(2) $$V_{\mathrm{WCA}}^{\mathrm{expand}}\left(r_{ij}\right)=4\epsilon \left[{\left(\frac{\sigma }{r_{ij}-\Delta_{ij}}\right)}^{12}-{\left(\frac{\sigma }{r_{ij}-\Delta_{ij}}\right)}^{6}\right]$$

where $\Delta_{ij}=\left(d_{i}+d_{j}\right)/2-2^{1/6}\sigma$, where $d_{i}$ and $d_{j}$ are the diamaters of particles *i* and *j*. The additional shift of $\Delta_{ij}$ by $2^{1/6}$ ensures the particle repulsion occurs for any $r_{ij}<\left(d_{i}+d_{j}\right)/2$. Interactions are truncated for $r_{j}>\Delta_{ij}+2^{1/6}\sigma$ to avoid attractions. The values of particles diameters are given in the Table 2.

### 2.4. Simulations and Lattice Preparation

We simulate the coarse-grained model via molecular dynamics (MD) calculations, carried out using the LAMMPS simulation suite [46]. To examine the stability of CD lattices, we first create defect free, fully bonded lattices. The lattice consists of 64 tetrahedral cages and 64 NPs, yielding a regular triclinic box (non-orthogonal with equal size lengths and an equal angle of 60${}^{\circ }$ between sides) of 64 primitive unit cells. Since the cubic diamond lattice can be considered as two identical inter-penetrating FCC lattices (with a shift of b/4 in each direction, where *b* is the FCC lattice constant), each particle type (tetrahedron or NP) is organized in an FCC lattice.

To create a CD lattice with DNA fully linked among NP and tetrahedra, we follow a two-step procedure. First, we simulate a single unit cell to form the links between the DNA strands connecting a NP and tetrahedron. We position the centers of the NP and tetrahedron at the ideal lattice positions for the CD structure (points (0,0,0) and (b/4, b/4, b/4) and link the center of the NP or tetrahedron to the lattice position via a strong harmonic potential. Since the NP is modeled as a dodecahedron (and each dodecahedron implicitly includes five co-centered tetrahedra), it is possible to create DNA links between one of these implicit tetrahedra of the dodecahedron and tetrahedral cage with no stress. The lattice constant *b* is chosen to geometrically allow for a connection between the NP and tetrahedron to form only along the nearest linking strand, preventing the formation of defects in this unit cell; this linked strand will not be stress free, but we will release this constraint later. We then perform a MD simulation of the primitive unit cell in a triclinic box with periodic boundary conditions in an NVT ensemble at temperature 0.09 (below the DNA melting temperature $T_{M}$). In the second step we replicate the fully bonded primitive cell generated in step 1 (with unwrapped periodic boundary conditions) to create a 4 by 4 by 4 lattice. We then remove the harmonic constraint on the central particles so they can freely move and simulate the lattice in an NPT ensemble at zero pressure and T=0.09 to ensure there is no external stress on the DNA linkers.

We use zero pressure since the osmotic pressure in experiments is very small. To study systems with different NP sizes, we change only the size of the isotropically functionalized NP, not the tetrahedral cage size, since NP size is more readily varied experimentally. We perform simulations for particle sizes ranging from d=40σ to 120σ, mapping to ≈26–78 nm in diameter. The simulations were performed for at least $4\times 10^{8}$ timesteps, where each timestep is 0.003. All of the parameters are given in LJ units. We use a Nose–Hoover thermostat and barostat in the simulation. The damping factors for thermostat and barostat are 1.0 and 3.0, respectively.

### 2.5. Surface Energy Calculation

To find the crystallite shape of the CD lattice, we use the Wulff construction method. This method requires the values of surface energy for different crystalline plane symmetries. To evaluate the surface energy of crystal facets with different symmetries, we must create lattices that are non-periodic in the direction normal to the facet under consideration. We use the same procedure employed for the periodic lattices just described to create fully bonded lattices with an exposed face. The system consists of a slab of diamond lattice that is periodic in the plane parallel to two lattice vectors, and non-periodic normal to the free surface (*z* direction). We again use P=0 after the initial defect-free lattice is formed. We consider three different symmetries of the exposed face: (100), (110), or (111). Depending on the symmetry of the lattice plane, we used either a cubic (100) or triclinic box (110 and 111). For triclinic boxes, the angle between *c* (the unit-cell vector in the *z* direction) and the other 2 unit-cell vectors is $90^{\circ }$; *a* and *b* (unit-cell vectors) lie in the xy plane. The size and angle between *a* and *b* are determined by the symmetry of plane. For the smallest system considered, the surface consists of 16 NPs, and the slab consists of eight layers transverse to the surface (four layers each of NP and tetrahedra). Each system consists of at least 128 NPs and tetrahedra in total. To check if our results are affected by the finite size of the slab, we performed simulations with six, eight, or ten layers of NPs for the 111 facet. The surface energy for different thicknesses was constant within the limits of uncertainty of the calculation, so we do not anticipate any finite size effects in our reported results.

## 3. Results & Discussion

### 3.1. Crystallite Shape from the Wulff Construction

To find the equilibrium shape of the crystallite, one should find the shape that minimizes the surface free energy of the crystallite for a given volume. The Wulff construction is a method to determine the equilibrium shape of a lattice symmetry based on the ratio between the surface energy γ and the distance *ℓ* between the crystallite center and the facet plane. Specifically, the ratio of the surface energy $\gamma_{hlk}$ of the facet normal to the hlk direction to the distance $\ell_{hlk}$ of that facet from the crystallite center should be constant for all facets with different symmetries. For each possible facet, one considers a plane, where the normal to the plane is in the (hkl) direction, and the distance from the center to the facet is *ℓ*. The subset of these planes that enclose the smallest volume around the center determine the shape of the crystallite.

(3) $$\lambda =\frac{\gamma_{hlk}}{\ell_{hlk}}$$

**Figure 2:**
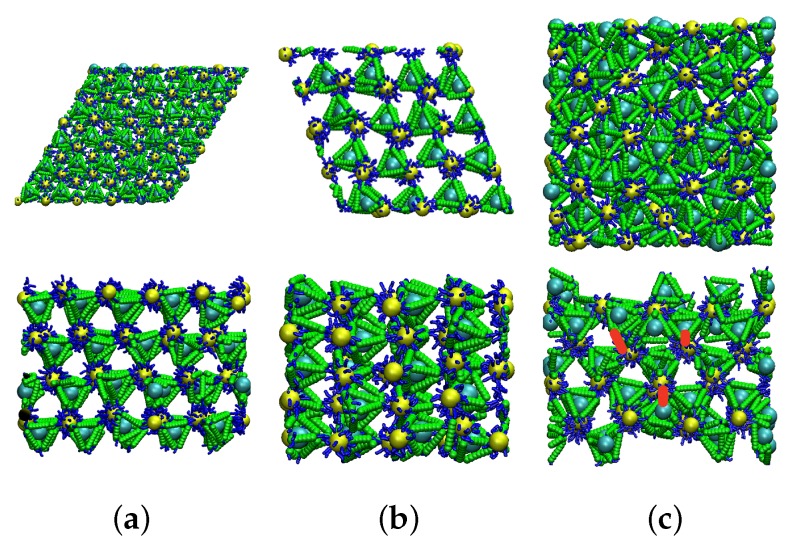
The snapshots of MD simulation of different finite crystal slabs with surfaces along different symmetry directions. (**a**) Top, the (110) surface, viewed normal to the free surface (*z*-direction); the bottom panel is a side view. (**b**) Top, the (111) plane, viewed normal to the free surface (*z*-direction); the bottom panel is a side view. (**c**) Top, the (100) plane, viewed normal to the free surface (*z*-direction); the bottom panel is a side view. The extra DNA bonds are highlighted by red lines.

We focus on surface energies of the (100), (110) and (111) surfaces. Since NP only interact with their nearest neighbors (short-ranged interaction), it is expected that the lower index planes are most important [47]. The snapshot of the simulated systems is shown in Figure 2. For a given facet symmetry, the suface energy will be determined by the number of broken links, which may depend of which of the possible parallel planes is exposed. This is because, in the ideal CD lattice, all of tetrahedral cages have the same orientation, but the opposite orientation from the NP, and all of the implicit tetrahedra of the NPs (dodecahedron) share the same orientation. The net result is that there are two possible scenarios, depending on the symmetry of the exposed face: (i) the number of broken bonds on the surface will (ideally) be 2, and thus surface energy does not depend on the position on the plane; or (ii) the number of broken bonds can be either 1 or 3 (ideally), depending on which parallel plane is exposed. We choose the plane that ideally leads to one broken bond, since this has lower energy. Because the tetrahedral cages and internal tetrahedra in NPs are in opposite directions, in order to have one broken bond on both sides of the slab, one surface will consist of tetrahedral cages, and the other will consist of NPs.

As described in the methods section, we can predict the surface energy of these interfaces from a simple “broken bond” model, assuming no distortion of the crystal structure, and compare this to a direct evaluation of surface energy from MD simulation, where interfaces may distort. In the broken bond approximation, we simply count the number of broken bonds per unit area for the chosen crystallographic plane and estimate the surface energy for each facet using the hybridization energy of DNA. This counting exercise predicts that the (111) face should have the lowest surface energy, and in this case, Wulff construction predicts the equilibrium crystallite is an octahedron, the same shape as atomic systems with diamond structure (e.g., diamond, Si, or SiO${}_{2}$) [40].

**Figure 3:**
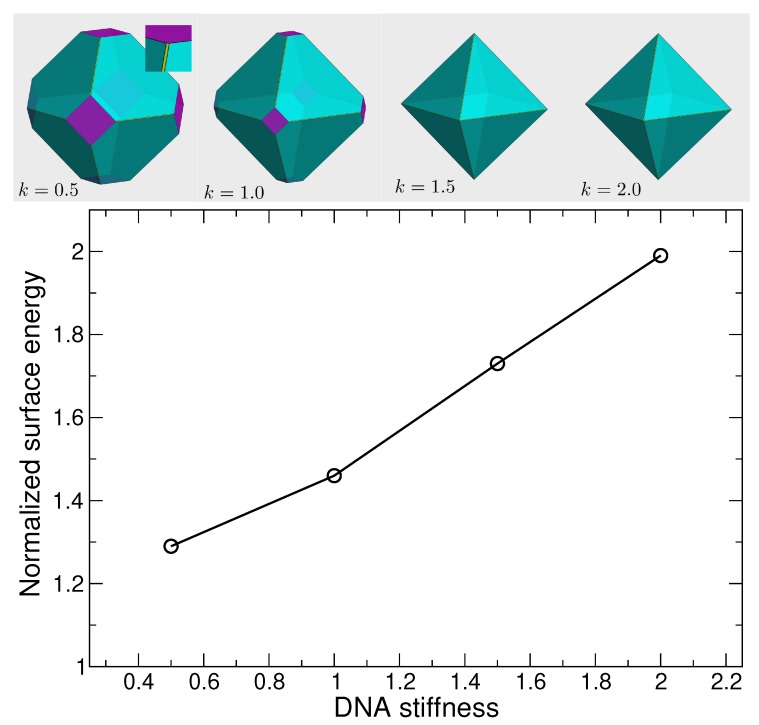
The surface energy of (100) plane as a function of DNA stiffness $k_{a}$ from MD simulations, and their corresponding crystallite shapes. The shapes were generated using WulffMaker [48]. The cyan, purple and yellow plane correspond to (111), (100) and (110) respectively. The (100) plane surface energy increases as the DNA stiffness increases; as a result the contribution of (100) plane in determining the crystal shape decreases. At the stiffness $k_{a}=2.0$, the crystallite shape is an octahedron. The contribution of (110) plane (the yellow planes) is not significant.

To test the validity of the predictions from the broken bond approximation, we evaluate the surface energy via MD simulations for slabs with facets of the same symmetries (100), (110), and (111) for NP diameter 40 and for variable values $k_{a}$ of the DNA stiffness. We define the surface energy

(4) $$\gamma_{hlk}=\frac{E_{hlk}-E_{\mathrm{bulk}}}{2A_{hlk}},$$

where the $E_{i}$ is the total energy of the simulated slab, $E_{\mathrm{bulk}}$ is the energy of bulk lattice, and $A_{i}$ is the area of exposed surface, and we express suface energies relative to the (111) plane so that we can easily compare to the broken bond approximation. We find that the ratio $\gamma_{110}/\gamma_{111}$ from the MD simulation (1.22) is nearly the same as predicted by the broken bond approximation (1.23), independent of the DNA stiffness. For the (100) plane, the broken bond model predicts the relative surface energy $\gamma_{100}/\gamma_{111}=1.73$, and as a result the (100) plane would have little impact on the crystallite shape. However, the MD simulations show that $\gamma_{100}/\gamma_{111}$ is sensitive to DNA stiffness (Figure 3), and can deviate significantly from the broken bond model prediction. For small stiffness, $\gamma_{100}/\gamma_{111}$ is substantially smaller than expected from the broken bond approximation, and approaches the broken bond prediction for large stiffness. We can understand the reduction of $\gamma_{100}/\gamma_{111}$ by the fact that flexible strands make it possible for some surface particles to reorient and form bonds with particles on the interior. Specifically, to create the (100) facet, we must break two of four bonds for each surface particle that were intact in the bulk system; for tetrahedra at the surface, they have enough flexibility at small $k_{a}$ to rotate towards NP in the interior layer, and create bonds with some dangling free strands of the NP. This is only possible since the interior NP have many more strands than are needed to create four bonds (the increase in the DNA strand density on the surface of NP increases the probability of such bonds). Thus, when the NP are on the surface and tetrahedra are interior, there will be no free DNA strands on the interior layer tetrahedra, since all tetrahedral cages inside the slab are already bonded. Thus, for the (100) direction, we create interfaces with tetrahedral cages on both sides of the slab, since this bonding through surface reorientation leads to a lower surface energy. See Figure 2c for an illustration of this phenomenon.

The variation in the surface energy $\gamma_{100}/\gamma_{111}$ with DNA stiffness implies variability in the crystallite shape, using the Wulff method. Given that $\gamma_{110}/\gamma_{111}=1.22$ independent of stiffness, the Wulff construction predicts that the crystallite shape will vary from a simple cube (for $\gamma_{100}/\gamma_{111}≲0.57$) to a simple octahedron (for $\gamma_{100}/\gamma_{111}≳1.73$). Figure 3 illustrates the expected crystallite shapes for the four stiffness values examined. At the smallest $k_{a}=0.5$, the shape is an octahedron (111) (cyan) truncated at the corners by a cube (100) (purple). As $k_{a}$ increases, the truncation by the octahedron increases, such for $k_{a}\geq 1.5$, the shape is essentially octahedral, though a careful inspection shows that the (110) plane (yellow) does slightly truncate the edges. This is also the shape predicted by the broken bond model. The importance of the (111) and (100) planes in the crystallite shape of DNA-functionalized NP lattices with diamond structure has been observed experimentally [31].

Exploiting the possibility of surface reorientation offers a path to control the crystallite shape. One approach to affect the reorientation of particles, and consequently crystallite shape, is to change the DNA stiffness as done here. Another possible approach is to use the DNA origami method to alter the geometry of the cages, and thereby change the bonding ability of cages and NPs. The difference in the number of possible DNA linking locations between the tetrahedra (four) and NP (20), coupled with sufficient flexibility of the DNA, results in a more stable system (lower surface energy) when tetrahedral cages are on the surface than when NPs are on the surface. Such increased stability should make the crystallite more resistant to heterogeneous melting at the interface.

In this work, we have not studied the effect of DNA length on the stability of the lattice. The importance of the DNA length on the stability and the quality of long-range order of the DNA-origami cages lattices has been seen experimentally [24]. Furthermore, the effect of DNA length on the stability of DNA-functionalized NP has been studied before [45].

### 3.2. Lattice Stability

#### 3.2.1. Lattice Structure

**Figure 4:**
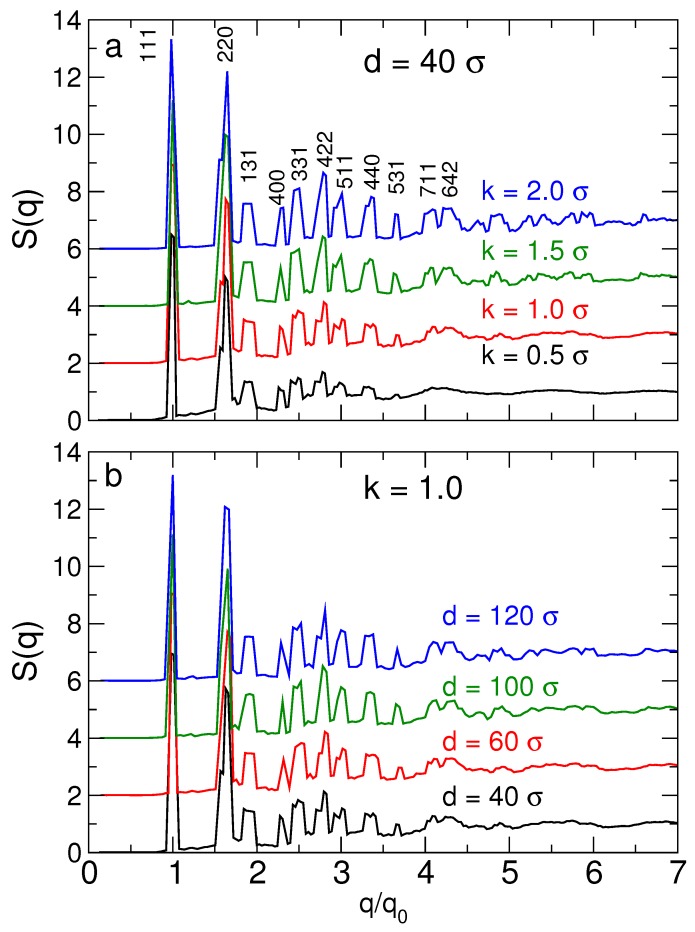
The structure factor S(q) for the cubic diamond lattice. (**a**) The structure factor for different DNA stiffness values. We label peaks by their corresponding Miller indices, known for the cubic diamond lattice. For stiffer DNA, the specific peaks of S(q) can be discerned for larger values of $q/q_{0}$ than for flexible DNA. In this sense, the stiffer DNA results in a better structured lattice. (**b**) The structure factor for different NP sizes. The effect of changing NP size on the structure is less pronounced than changing the DNA stiffness. For clarity of the figure, data are shifted vertically.

Before we calculate the stability from the relative free energy, we provide a reference for the effects of changing NP size and DNA stiffness on the crystal structure. To do so, we evaluate the structure factor

(5) $$S\left(q\right)=\frac{1}{N}\langle \sum_{j\neq k}^{N}e^{-iq\cdot (r_{j}-r_{k})}\rangle$$

where $r_{j}$ is the position of the center of a NP or tetrahedral cage. Figure 4 shows S(q) for diamond lattices for representative particle sizes and DNA backbone stiffness values; the stiffness (or persistance length) is directly linked to salt concentration. For all systems, the simulated S(q) reproduces the theoretically expected locations for at least the first 6 peaks of S(q), confirming the mechanical stability of the cubic diamond lattice. The higher order peaks become “smeared” due to lattice vibrations. Figure 4a shows that increasing the DNA stiffness (equivalent to lower salt concentration) results more sharply defined peaks, which are discernable up to larger *q* values. For example, for stiffer DNA ($k_{a}=2.0$), we can distinguish two peaks near $q/q_{0}=4.2$, which for more flexible DNA ($k_{a}=0.5$) combine to single broad peak. Thus, as might be anticipated, increasing DNA stiffness results in a more precisely defined structure of the lattice. Figure 4b shows that the lattice structure is only weakly affected by the NP size. The challenge is to understand the balance between optimizing the precise structure of the lattice and its relative stability.

#### 3.2.2. Lattice Vibrational Spectrum

Since we are dealing with lattice systems, their free energy will be determined from the lattice vibrational spectrum. In principle, we could directly evaluate the dispersion relation ω(q), but the size of the system (up to 48896 force sites) and complexity of the potentials make the necessary matrix diagonalization computationally infeasible. Thus, we need to consider a simplified representation of the system. We expect that the vibrational spectrum is primarily encoded in the vibrational properties of the centers of mass of the NP and tetrahedra. Thus, we can coarse-grain the vibrational motion of the complete system from our MD simulations to the vibrations of the centers of mass of the NPs and tetrahedral cages; the mass of these sites corresponds to the total mass of NPs and tetrahedral cages (including DNA strands) from the MD simulation. To obtain the dispersion relation, we use the dynamical matrix approach [49,50,51,52,53]. The dynamical matrix

(6) $$D_{\mu \nu }^{-1}\left(q\right)=\frac{1}{k_{B}T}\langle u_{\mu }\left(q\right)u_{\nu }\left(q\right)\rangle$$

is defined by the correlation function of the Fourier transforms of the NP and tetrahedral coordinates $u_{\mu }\left(q\right)$ and $u_{\nu }\left(q\right)$ [54,55].

The dynamical matrix can be related to the normal mode dispersion relation by assuming a simplified harmonic model for the interactions among the lattice sites [49,50]. Specifically, one assumes a simple potential between lattice sites

(7) $$V=\frac{1}{2}k_{r}\sum_{n}\delta r_{ij}^{2}+\frac{1}{2}r_{0}k_{\theta }\sum_{n}\delta \theta_{ijk}^{2},$$

where we sum *n* over all of unit-cells, $\delta r_{ij}$ is the distance between particles *i* and *j* relative to the ideal separation $r_{0}$, and $\delta \theta_{ijk}$ is the angle (relative to its ideal value) between particles *i* and two nearest neighbors *j* and *k*. The radial bond constant $k_{r}$ mimics the elasticity of the DNA bond between NPs and tetrahedral cages, and $k_{\theta }$ is related to the rotational degrees of freedom of sites around their centers. The constants $k_{r}$ and $k_{\theta }$ will be determined from the dynamical matrix. Specifically, the Fourier transform of the equation of motion for the harmonic approximation to the potential can be written as

(8) $$\omega^{2}\;u_{\mu ,\alpha }\left(q\right)=\sum_{\nu ,\beta }D_{\mu \nu }^{\alpha \beta }\left(q\right)\;u_{\nu ,\beta }\left(q\right),$$

where ω is the frequency of vibration at wave vector *q*, μ and ν are particle indices, and α and β are directions.

**Figure 5:**
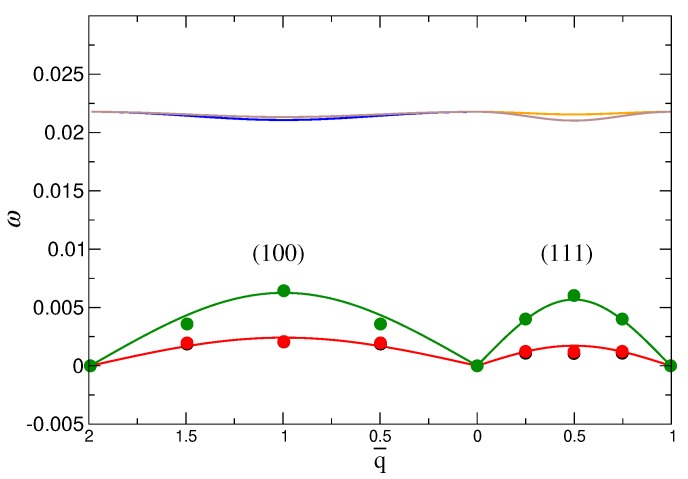
The dispersion relation obtained from simulation and fit with theory [51] for the (100) and (111) directions. The points are the values calculated from MD simulation based on the eigenvalues of the dynamical matrix (Equation (8)). The solid line is the fit with the dispersion form given in ref. [51]. These data are for the system with DNA stiffness of $k_{a}=1.0$ and NP size d=40σ. $\bar{q}=qb/2\pi$ is the reduced wave vector and *b* is the lattice constant.

We directly calculate these frequencies ω using the dynamical matrix from the eigenvalue problem of Equation (8). Since we have coarse-grained our simulation lattice to a 4×4 lattice of NP and tetrahedra, we are limited to just four different wave vectors *q* for each of six branches, the number of which is determined by having two particles in each unit-cell. The symbols in Figure 5 shows the calculated ω for the three lowest branches along the the most common symmetry directions, (100) and (111); there are only two sets of points, since the two lowest branches are degenerate [51]. We focus on the lowest branches because they have the most significant contribution to the free energy. Since the dynamical matrix is related to the potential of Equation (7), its eigenvalues are functions of $k_{r}$ and $k_{\theta }$, and we can use our results to estimate these parameters, and thus estimate the free energy. Based on data for the two directions, (100) and (111), accounting for degeneracy, and that the origin is a common point among the symmetry directions, we have a total of 13 unique points to determine $k_{r}$ and $k_{\theta }$. The solid lines in Figure 5 show the resulting fit for the dispersion relation, which matches well with the available data. We will use these fit values in the next section when we evaluate the free energy.

#### 3.2.3. Lattice Free Energy

We now proceed to evaluate the relative thermodynamic stability as a function of particle size and DNA stiffness. To do so, we need to evaluate the change in Gibbs free energy ΔG=ΔE−TΔS+PΔV, as a function of particle size or DNA stiffness. The smallest particle size (diameter 40σ) and smallest stiffness ($k_{a}=0.5$) is chosen as the reference state, and the free energy for all other sizes is evaluated relative to that system. Since we use P=0, the PΔV term is not needed, and the Gibbs free energy reduces to Helmholtz free energy.

To evaluate the lattice free energy, the Hamiltonian can be written in terms of the normal coordinates of vibration in Fourier space,

(9) $$H\left(q,\lambda \right)=|P_{\lambda }{\left(q\right)|}^{2}+\omega_{\lambda }^{2}\left(q\right){\left|Q_{\lambda }\left(q\right)\right|}^{2},$$

where $P_{\lambda }$ and $Q_{\lambda }$ are normal coordinates, and λ indicates different branches of the dispersion relation. Based on this Hamiltonian, the Helmholtz free energy

(10) $$F=-k_{B}T\ln\left(\sum_{\lambda }\int e^{-\beta H(q,\lambda )}\;dq\right)$$

**Figure 6:**
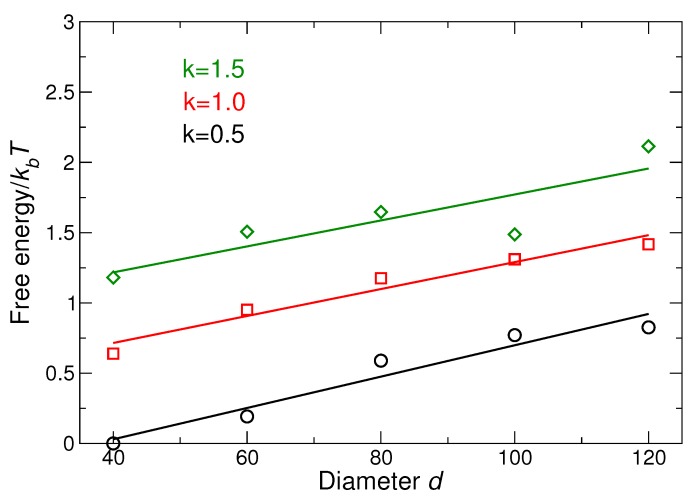
Difference in free energy of the lattice for different DNA stiffness values as a function of NP size; the reference system is the system with smallest NP size and $k_{a}=0.5$. Increasing NP size increases the free energy, which means a relative decrease in stability. Similarly, increasing stiffness increases free energy (reduces stability).

We have already numerically and analytically [51] evaluated the dispersion along the (100) and (111) directions, and we can use the resulting fit values of $k_{r}$ and $k_{\theta }$ to numerically calculate the free energy when we integrate over all directions; the result is shown in Figure 6.

Our lattice free energy data (Figure 6) show that the relative free energy increases with increasing NP size and with increasing DNA strand stiffness. In other words, the larger NP or stiffer strands have a decreased relative thermodynamic stability. Recall that increasing stiffness results in a more precisely defined diamond structure (Figure 4); thus, a balance must be struck between optimizing the structure through DNA stiffness and the relative thermodynamic stability. The decrease in stability of lattices as the NP size increases for faced-center cubic and body-centered cubic lattices has been seen in previous study [56] that is consistent with our findings for CD lattice. The effect of DNA stiffness in the context of DNA grafting density (number of DNA linkers connecting the two NPs) has been studied previously [57]. However, changing the number of linkers also directly changes the enthalpy of the system. In the current study, all of the DNA linkers on the tetrahedral cage are connected to NPs. Thus, increasing the grafting density does not change the number of bonded DNA linkers, as a result, the effective stiffness of the connections between particles is independent of the grafting density.

From a design perspective, it is helpful to know how different aspects of the lattice vibrations affect the free energy. For example, how much of the stability originates from energetic versus entropic effects? The free energy is separable into energetic and entropic contributions to ΔF=ΔE−TΔS. The energy can be extracted directly from simulation the data, and ΔE is essentially independent of NP size and stiffness. This is due to the fact that there are almost no non-bonded interactions with NP, unless NP are so large that they overlap. Additionally, DNA bonding does not vary with NP size, so the DNA do not significantly contribute to ΔE. Consequently, entropy is the controlling factor in the free energy.

**Figure 7:**
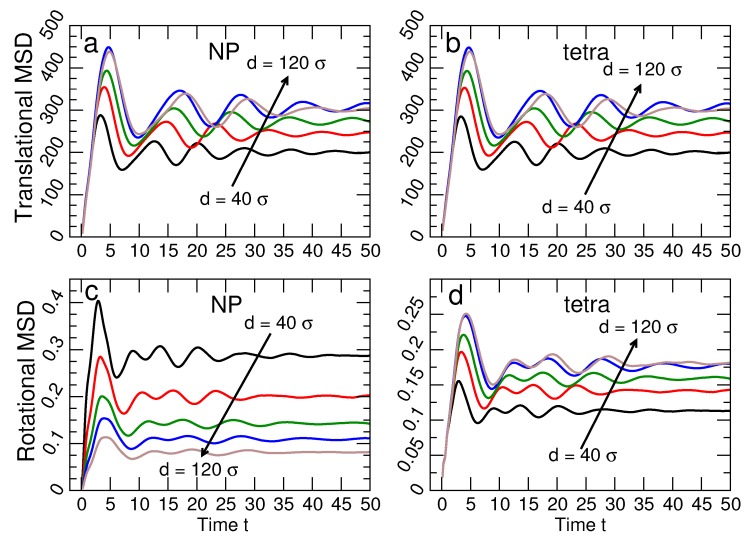
The time dependence of the translational and rotational vibration of the tetrahedral cages and NPs. The translational mean-squared displacement $\langle r^{2}\left(t\right)\rangle$ for (**a**) NP and (**b**) tetrahedra are very similar; the saturation value $\langle u^{2}\rangle$ of the MSD increases for both NP and tetrahedra with increasing NP size. The rotational mean-squared displacement $\langle \theta^{2}\left(t\right)\rangle$ for (**c**) NP and (**d**) tetrahedra differ significantly. Specifically, the saturation value $\langle \phi^{2}\rangle$ for NP decreases as the NP size increases, while $\langle \phi^{2}\left(t\right)\rangle$ for tetrahedral cages increases as the NP size increases. Note that dependence on NP size of the rotational MSD of NPs is different than the other three MSDs. All data are for the system with stiffness $k_{a}=1.0$. The black, red, green, blue and brown lines correspond to d=40,60,80,100,120σ, respectively.

We wish to parse the entropy into the contributions from the rotational and vibrational degrees of freedom. In our dynamical matrix approach, the free energy (entropy) is a complicated function of $k_{r}$ and $k_{\theta }$, and is defined only numerically when we integrate over all lattice directions. This makes it difficult to study the effects of rotational and vibrational degrees of freedom separately. Although we know intermolecular correlations contribute to the free energy, it is nonetheless useful to consider a simple mean-field description that captures the essential features of the free energy from the more complex dynamical matrix calculation. Specifically, we ignore correlations of the vibrations, and consider each NP and tetrahedron as an independent oscillator. In this independent harmonic approximation,

(11) $$V=\sum_{i=1}^{N_{\mathrm{NP}}}\left(\frac{1}{2}k_{\mathrm{trans}}^{\mathrm{NP}}\Delta r_{i}^{2}+\frac{1}{2}k_{\mathrm{rot}}^{\mathrm{NP}}\Delta \theta_{i}^{2}\right)+\sum_{i=1}^{N_{\mathrm{tet}}}\left(\frac{1}{2}k_{\mathrm{trans}}^{\mathrm{tet}}\Delta r_{i}^{2}+\frac{1}{2}k_{\mathrm{rot}}^{\mathrm{tet}}\Delta \theta_{i}^{2}\right),$$

where $\Delta r_{i}^{2}$ is the mean-square translational vibration of the centers of NP or tetrahedra, and $\Delta \theta^{2}$ is the mean-square angular vibration. In this harmonic representation, the force constants $k_{\mathrm{trans}}$ and $k_{\mathrm{rot}}$ can be determined from the typical size of the lattice vibrations. $k_{\mathrm{trans}}$ and $k_{\mathrm{rot}}$ are related to, but distinct from $k_{r}$ and $k_{\theta }$. Figure 7 shows the average time dependence of $\langle r^{2}\left(t\right)\rangle$ and $\langle \theta^{2}\left(t\right)\rangle$ for both the tetrahedra and the NP for many NP sizes at $k_{a}=1.0$; θ(t) is the angle between the vector defined from the center of a NP or tetrahedron to a chosen vertex at times $t_{0}$ and $t_{0}+t$ (where $t_{0}$ is an arbitrary initial time).

**Figure 8:**
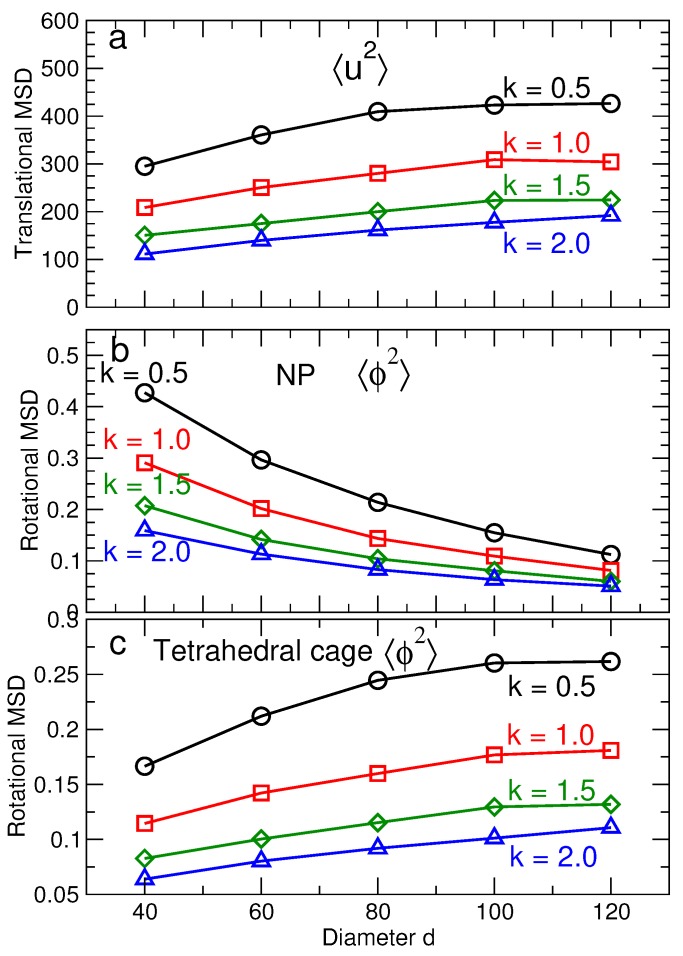
(**a**) The characteristic translational MSD $\langle u^{2}\rangle$ of the NPs and tetrahedral cages as a function of NP size for different DNA stiffness. (**b**) The characteristic rotational MSD $\langle \phi^{2}\rangle$ of NPs as a function of NP size for different DNA stiffness. This decrease in $\langle \phi^{2}\rangle$ is the main factor decreasing the stability the CD lattice. (**c**) The rotational MSD $\langle \phi^{2}\rangle$ of tetrahedral cages as a function of NP size for different DNA stiffness values. The translational and rotational MSD decreases as the stiffness increases. The black, red, green and blue lines correspond to $k_{a}=0.5,1.0,1.5,2.0$ respectively.

The vibrational amplitudes saturate at characteristic values $\langle u^{2}\rangle$ for translations and $\langle \phi^{2}\rangle$ for rotations for $t≳10^{4}$. In other words, lattice particles vibrate around fixed positions, with no diffusion of the NPs or tetrahedra. Note that Figure 7a,b shows that $\langle u^{2}\rangle$ is essentially the same for NP and tetrahedra, while Figure 7c,d shows that $\langle \phi^{2}\rangle$ differs for NP and tetrahedra. To show these trends more clearly, Figure 8 shows $\langle u^{2}\rangle$ and $\langle \phi^{2}\rangle$ for all NP sizes and DNA stiffness values. For the translational component $\langle u^{2}\rangle$, we average the contribution from the NP and tetrahedra, since the values for each are essentially identical. In contrast, the rotational displacement $\langle \phi^{2}\rangle$ is significantly different for NP and tetrahedra. Specifically, $\langle \phi^{2}\rangle$ of NP decreases with increasing NP diameter, while $\langle \phi^{2}\rangle$ increases for tetrahedral cages as the NP diameter increases. The opposite behavior of the angular displacement can be understood geometrically. Specifically, we can relate $\langle \phi^{2}\rangle$ to an arc length *s* via $\langle \phi^{2}\rangle \approx {\left(2s/d\right)}^{2}$ where *d* is the diameter of the NP or tetrahedron. Since the NP and tetrahedra are connected, the arc length *s* must be the same for either particle type. Thus, as NP diameter increases, $\langle \phi^{2}\rangle$ will decrease for NP, even as it grows for tetrahedra.

Based on the independent oscillator approach, the relative entropy as a function of NP size or DNA stiffness can be separated into translational and rotational degrees of freedom. In this approximation, $k_{\mathrm{trans}}=3k_{b}T/\langle u^{2}\rangle$, and the translational contribution to the entropy is

(12) $$\Delta S_{\mathrm{trans}}=\frac{3}{2}k_{B}\left[\ln{\left(\frac{\langle u^{2}\rangle }{\langle u_{\mathrm{ref}}^{2}\rangle }\right)}_{\mathrm{NP}}+\ln{\left(\frac{\langle u^{2}\rangle }{\langle u_{\mathrm{ref}}^{2}\rangle }\right)}_{\mathrm{tetra}}\right].$$

The reference $\langle u_{\mathrm{ref}}^{2}\rangle$ corresponds to the system with a NP diameter 40σ. In principle, Equation (12) should also contain a $\frac{T}{\langle u^{2}\rangle }\frac{d\langle u^{2}\rangle }{dT}$ term, but this term can be eliminated based on the *T* dependence of $\langle u^{2}\rangle$; specifically, simulations show that the $\langle u^{2}\rangle$ depends linearly on temperature (as would be expected for nearly harmonic vibrations), provided we do not closely approach the melting temperature. Linear *T* dependence of $\langle u^{2}\rangle$ implies that $\frac{T}{\langle u^{2}\rangle }\frac{d\langle u^{2}\rangle }{dT}=1$, and independent of particle size. Similar to the translational component, $k_{\mathrm{rot}}=3k_{b}T/\langle \phi^{2}\rangle$, and the relative entropy from the rotational degrees of freedom is given by

(13) $$\Delta S_{\mathrm{rot}}=\frac{3}{2}k_{B}\left[\ln{\left(\frac{\langle \phi^{2}\rangle }{\langle \phi_{\mathrm{ref}}^{2}\rangle }\right)}_{\mathrm{NP}}+\ln{\left(\frac{\langle \phi^{2}\rangle }{\langle \phi_{\mathrm{ref}}^{2}\rangle }\right)}_{\mathrm{tetra}}\right]$$

**Figure 9:**
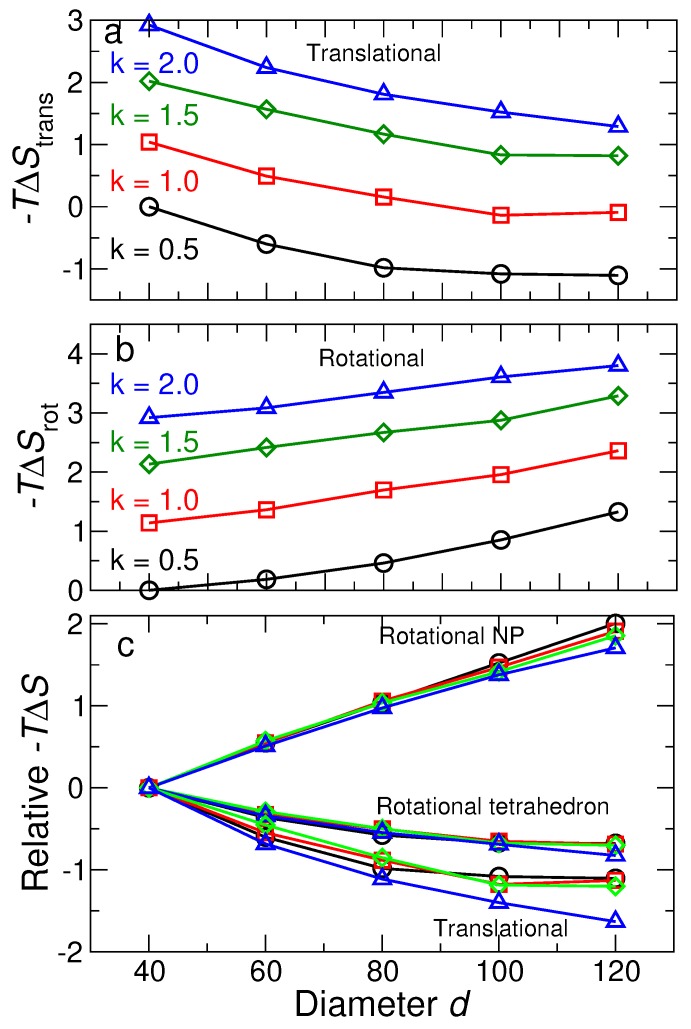
(**a**) The change in the translational free energy $-T\Delta S_{\mathrm{trans}}$ as a function of NP diameter for many stiffness values. The free energy is reported relative to the reference system (d=40σ and stiffness $k_{a}=0.5$). (**b**) The change in the total rotational free energy $-T\Delta S_{\mathrm{trans}}$. (**c**) The translational free energy and rotational free energy of NPs and tetrahedral cages as a function of NP size. Different stiffness values are plotted relative to the smallest NP with the same stiffness, to eliminate shifts of free energy due to changes of stiffness. The systems with different stiffness have the same behavior as a function of NP size, suggesting that the DNA stiffness just shifts the free energy by a constant.

Based on the values obtained for $\langle u^{2}\rangle$ and $\langle \phi^{2}\rangle$, we show the resulting contributions to the free energy from the translational and rotational degrees of freedom for NP and tetrahedra (Figure 9). The contribution to the entropy from translational degrees of freedom is the same for tetrahedra and NP, so we do not show them separately; Figure 9a shows that $-T\Delta S_{\mathrm{trans}}$ *decreases* with increasing NP size. Figure 9b shows that the rotational contribution $-T\Delta S_{\mathrm{rot}}$ has to opposite dependence on NP size—*increasing* free energy with increasing size; we can further dissect the rotational contribution from NP and tetrahedra. For tetrahedra, $-T\Delta S_{\mathrm{rot}}$ shows the same qualitative behavior as $-T\Delta S_{\mathrm{trans}}$. In contrast, $-T\Delta S_{\mathrm{rot}}$ for NP decreases with increasing NP size. The reason for opposite behavior of the rotational entropy of NP and tetrahedra is precisely the reason for the difference in $\langle \phi^{2}\rangle$, discussed previously. Given the overall increase in the free energy with NP size, it is apparent that the dominant contribution to the free energy comes from rotations of the NP. Thus, this is a natural term to target if one wishes to affect the relative stability of diamond NP lattices.

The qualitative effects of DNA stiffness (determined experimentally from salt concentration) are the same for all components: increasing stiffness decreases the entropy, and thus, increases free energy. Moreover, the quantitative variation of the free energy with stiffness is simpler than is apparent in Figure 9a,b. To illustrate this fact, we re-plot the contributions to the entropy for each stiffness value relative to the entropy of the smallest NP with the same stiffness. Figure 9c shows that, when shifted this way, the size dependence of the entropy is the same for all stiffness values. Thus, the effect of the stiffness, at least over the range we have studied, is to introduce a simple shift of the entropy.

## 4. Conclusions

We studied the crystallite shape of the CD lattice. The specific design of the DNA-origami cage and its effect on the number of bond sites leads to a reduction in the surface energy of specific symmetries, and consequently alters the equilibrium crystallite shape. Specifically, the predicted shape of crystallite for CD lattices without surface reorientation is an octahedron, which shifts to a cube-truncated octahedron when surface reorientation is possible. We also have studied the effects of NP size and DNA stiffness on the stability of the CD lattices. We found that the increase of stiffness and NP size (in the range of this study) increase the free energy and consequently decrease the relative thermodynamic stability of these lattices. On the other hand, the increase of stiffness improves the structure of these lattices. So there is a potential trade off between having a well structured lattice or a more stable lattice. The less stiff DNA leads to a more stable system, and this effect has seen previously in the patchy particles systems [58]. By separating the free energy into parts, we demonstrated that the rotational degrees of freedom of NPs are the dominant factor reducing lattice stability as the NP size increases, while the other degrees of freedom (rotation of tetrahedra and translational degrees of freedom of both types of particles) stabilize it.

## Figures and Tables

**Table 1:** The bond potential parameters for the tetrahedral cage and NP force sites. For the NP bonds Δ=d/2−0.96, where *d* is NP diameter and 0.96 is the minimum of FENE potential when Δ=0, which insures that the minimum of bond potential is at d/2. The distance between vertices of dodecahedron is l=d/2.8, where d is the dodecahedron circumscribed sphere’s diameter.

Particle Type	Bond	$k_{b}$	$R_{0}$	ϵ	Δ
Tetrahedral cage	Edge beads, first neighbor	240	1.5	8	6.36
	Edge beads, second neighbor	240	1.5	8	13.67
	Vertex to center	240	1.5	8	36.52
	Vertex to edge	30	1.5	1	15.08
	Edge to edge	30	1.5	1	9.15
Nanoparticle	Center to vertex	30	1.5	1	$\frac{d}{2}-0.96$
	Vertex to vertex	30	1.5	1	l−0.96

**Table 2:** The particle diameter for excluded volume interactions given by Equation (2). For details about the interactions between DNA sites see ref. [43].

Particle Type	Diameter (σ)
Tetrahedral cage, edge particles	9.9
Tetrahedral cage, central particle	40
NP, central particle	ranging between 40 to 120
DNA backbone	1
